# *IL-6* gene amplification and expression in human glioblastomas

**DOI:** 10.1054/bjoc.2001.1942

**Published:** 2001-08

**Authors:** A Tchirkov, C Rolhion, S Bertrand, J-F Doré, J-J Dubost, P Verrelle

**Affiliations:** Centre Jean Perrin, 58 rue Montalembert, Clermont-Ferrand, 63011, France; Faculté de Médecine, Laboratoire de Cytogénétique, place Henri Dunant, Clermont-Ferrand, 63001, France; INSERM U484 rue Montalembert, Clermont-Ferrand, 63005, France; Centre Léon Bérard, 28 rue Laënnec, Lyon, 69373, France; Unité d’Immunologie Clinique, Service de Rhumatologie, CHU – Hôpital Gabriel Montpied, Clermont-Ferrand, 63003, France

**Keywords:** *IL-6*, gene amplification and expression, glioblastoma

## Abstract

The aggressiveness of human gliomas appears to be correlated with the upregulation of interleukin 6 (*IL-6*) gene. Using quantitative PCR methods, we detected amplification and expression of the *IL-6* gene in 5 of 5 primary glioblastoma samples and in 4 of 5 glioblastoma cell lines. This finding suggests that the amplification of *IL-6* gene may be a common feature in glioblastomas and may contribute to the *IL-6* over-expression. © 2001 Cancer Research Campaign http://www.bjcancer.com


*British Journal of Cancer* (2001) **85**(4), 518–522
© 2001 Cancer Research Campaign
doi: 10.1054/ bjoc.2001.1942, available online at http://www.idealibrary.com on http://www.bjcancer.com

### *IL-6* gene amplification and expression in human glioblastomas

**A Tchirkov[1,2], C Rolhion[1,3], S Bertrand[4], J-F Doré[4], J-J Dubost[5] and P Verrelle[1,3]**

[1]Centre Jean Perrin, 58 rue Montalembert, 63011 Clermont-Ferrand, France; [2]Laboratoire de Cytogénétique, Faculté de Médecine, place Henri Dunant, 63001 Clermont-Ferrand, France; [3]INSERM U484, rue Montalembert, 63005 Clermont-Ferrand, France; [4]Centre Léon Bérard, 28 rue Laënnec, 69373 Lyon, France; [5]Unité d'Immunologie Clinique, Service de Rhumatologie, CHU – Hôpital Gabriel Montpied, 63003 Clermont-Ferrand, France

**Summary** The aggressiveness of human gliomas appears to be correlated with the upregulation of interleukin 6 (*IL-6*) gene. Using quantitative PCR methods, we detected amplification and expression of the *IL-6* gene in 5 of 5 primary glioblastoma samples and in 4 of 5 glioblastoma cell lines. This finding suggests that the amplification of *IL-6* gene may be a common feature in glioblastomas and may contribute to the *IL-6* over-expression. © 2001 Cancer Research Campaign http://www.bjcancer.com

**Keywords**: *IL-6*; gene amplification and expression; glioblastoma

The release of biologically active interleukin 6 (*IL-6*) frequently occurs in human glioblastomas, the most malignant brain tumours (Van Meir et al, 1990). In our previous study on a large series of 59 gliomas, we have demonstrated a strong association between the aggressiveness of glial tumours and degree of *IL-6* expression at the RNA level (Rolhion et al, 2001). As *IL-6* has properties of a growth factor for tumour cells (Goswami et al, 1998) and is able to block apoptosis induced by various chemotherapeutic compounds (Borsellino et al, 1995) or reactive oxygen species (Miwa et al, 2000), the expression of the *IL-6* gene may be one of the most important factors contributing to the growth and survival of glioblastoma cells in patients under radiotherapy and chemotherapy.

The mechanism underlying the *IL-6* upregulation in glioblastoma still remains unclear. The results of molecular cytogenetics in glioblastoma point to frequent gains of chromosome 7 and amplification events on 7p (Weber et al, 1996). Since the *IL-6* gene is located on 7p21, the up-regulation of *IL-6* expression in tumour cells could be mediated by amplification of the gene. In order to test this hypothesis, we determined the number of *IL-6* gene copies and *IL-6* mRNA levels in glioblastoma cell lines and primary cells using quantitative PCR assays.

### MATERIAL AND METHODS

### Tumour material

Five glioblastoma cell lines including SF763 and SF767 (Joy et al, 1997), U87 and U251 (van Meir et al, 1994) and G5 (Buronfosse et al, 1994) were used in this study. In addition, five samples of primary tumour cells were obtained from patients with glioblastoma who underwent surgery.

*Received 10 November 2000*
*Revised 28 March 2001*
*Accepted 30 May 2001*

*Correspondence to:* C Rolhion

### DNA and RNA extraction, cDNA preparation

DNA and total RNAs were extracted using Trizol® reagent (GIBCO/BRL, Grand Island, NY), according to the manufacturer's instructions. Reverse transcription of RNA was performed as described elsewhere (Rolhion et al, 1999).

### Competitive *IL-6* gene dosage assay

The *IL-6* competitor was produced from normal human genomic DNA using the PCR amplification method with a composite primer (Celi et al, 1993). The conventional and composite *IL-6* primers were derived from the *IL-6* gene sequence (Yasukawak et al, 1987) as follows: ILF, 5′-AATTCGGTACATCCTCGACGG-3′ (forward); ILR, 5′-GGGCATGGATTTCAGACCC-3′ (reverse); ILAILR, 5′-GGGCATGGATTTCAGACCCCAGTCTAG-GTCGTTGGCCTCA-3′ (composite primer). Schematic diagram illustrating the generation of the competitor is presented in Figure 1A. Constant amounts of target DNA and serially diluted competitor were co-amplified using the ILF/ILR primers. After the separation on a 2%-agarose gel, the yields of amplified competitor and target products were analysed and quantified using the Imager Gel Documentation System (Appligene Oncor, Illkirch, France). The amount of *IL-6* molecules in the sample per nanogram of DNA was calculated from the equivalence point where the amount of amplified target was equal to the amount of amplified *IL-6* competitor (Figure 1B). The relative changes in the number of *IL-6* copies in tumour DNA were estimated in comparison with normal human DNA used as a reference in each PCR experiment (Figure 1C).

### Real-time PCR analysis of gene dosage

We quantitated *IL-6* gene dosage using real-time PCR in the LightCycler system (Roche Diagnostics, Meylan, France). The amplification of *IL-6* gene was performed using ILF/ILR primers and a DNA Master SYBRGreen I reagent set (Roche Diagnostics).

A Tchirkov and C Rolhion contributed equally to this work.

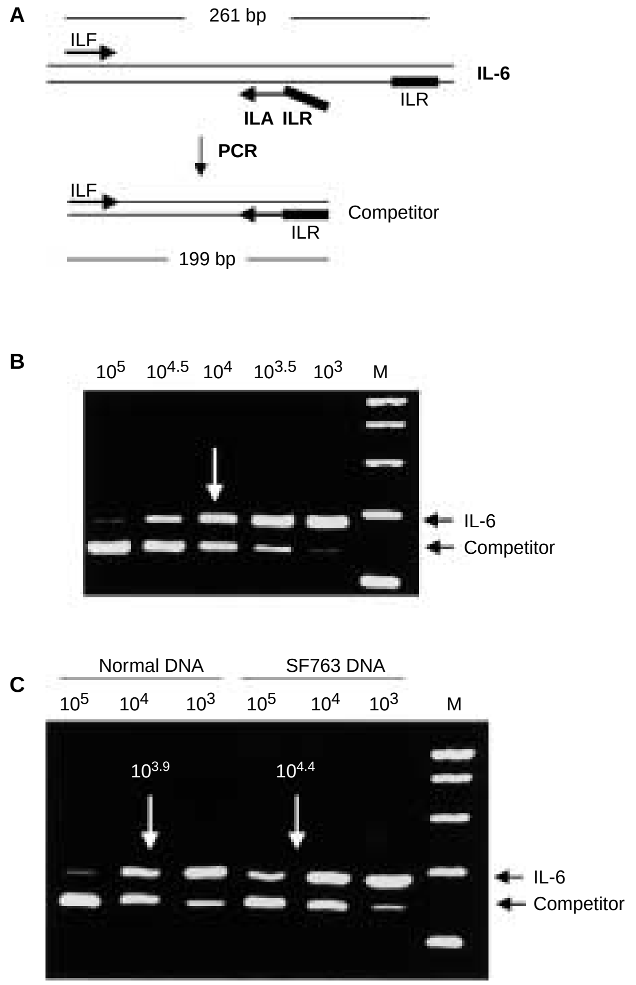

**Figure 1** (**A**) Construction of the *IL-6* DNA competitor. Note that the 3′ end of the composite primer ILAILR (segment ILA) corresponds to the opposite strand of the target sequence at a predetermined distance from the primer ILF, and the 5′ end is identical to the sequence of the primer ILR, whose binding site is located 43 bp upstream from the segment ILR. (**B**) Co-amplification of serial dilutions of competitor with a constant amount of normal human DNA using ILF/ILR primers, spanning exon 2/intron 2 of the *IL-6* gene. The number of competitor molecules added is shown above each lane. Two amplification products are generated, one derived from the target DNA (*IL-6*) and another one, 62 bp smaller, derived from the competitor (COMP). The equivalence point, at which competitor and target bands would have the same intensity, is indicated by an arrow. M, PCR markers (Promega). (**C**) Quantitation of *IL-6* gene copies present in control and glioblastoma (SF763 cell line) DNA samples using competitive PCR. The equivalence points between the competitor and sample bands corresponded to $10^{3.9}$ and $10^{4.4}$ competitor molecules as determined by means of densitometry. Compensation for the difference in size between the competitor and sample bands was made by multiplying the number of competitor molecules at equivalence point by 0.76. The amounts of DNA used for PCR being known, the numbers of competitor molecules at equivalence point were converted into the numbers of *IL-6* molecules per ng DNA: 221 for normal DNA sample and 633 for SF763 DNA sample. M, PCR markers (Promega)

In parallel in the same samples, we assessed the dosage of another gene from chromosome 7, the cystic fibrosis transmembrane conductance regulator (CFTR) gene located on 7q31.2. The amplification of *CFTR* gene was performed using the primers described by Dean et al (1990). To provide a genomic reference point for each sample, the copy number of beta-globin gene, which is located on chromosome 11 (11p15.5), was determined. The region 11p15.5 has not been reported to be involved in chromosomal gains and losses observed in human glioblastomas (Schlegel et al, 1996; Nishizaki et al, 1998). The sequences of beta globin gene-specific primers were as follows: forward, 5′-GGTTGGCCAATC-TACTCCCAG-3′; reverse, 5′-GCTCACTCAGTGTGGCAAAG-3′. For dosage analysis of each gene, calibration curves were constructed with serial dilutions of purified specific PCR products which contained known numbers of DNA molecules. The results of real-time PCR were expressed as a ratio of the copy number determined for the target gene (*IL-6* or *CFTR*) to the copy number of the control gene, beta globin.

### Semiquantitative RT-PCR for *IL-6* mRNA with an endogenous standard

*IL-6* mRNA levels were assessed using a semiquantitative RT-PCR as described in details previously (Rolhion et al, 1999). In this approach, the ratio expression rate (RER) of *IL-6* to β2-microglobulin genes was determined.

### Real-time RT-PCR for *IL-6* mRNA

To quantitate *IL-6* mRNA, fluorescent TaqMan methodology and ABl Prism 7700 Sequence Detection System (Perkin Elmer Applied Biosystems, Courtaboeuf, France) were used. A relative quantitation was made with the comparative threshold cycles ($\Delta\Delta C_T$) method (PE User Bulletin #2, 1997) where the amount of *IL-6* target was normalized to an endogenous reference, the *18S rRNA* gene, and relative to a calibrator, the U251 glioblastoma cell line. The following amplification primers and probes were used: *IL-6* forward primer 5′-TGAACTCCTTCTCCACAAGCG-3′; reverse primer 5′-TCTGAAGAGGTGAGTGGCTGTC-3′ and labelled probe 5′-TGGTGTTGCCTGCTGTCCCT-3′; *18S* rRNA gene forward primer 5′-CGGCTACCACATCCAAGGAA-3′, reverse primer 5′-GCTGGAATTACCGCGGCT-3′ and probe 5′-TGCTG-GCACCAGACTTGCCCT-3′.

### RESULTS

### *IL-6* gene dosage

We first quantified the number of *IL-6* gene copies in DNA samples using competitive PCR. In 5 control human genomic DNA samples, this method determined a mean number of 226 (± 45, SD) *IL-6* gene molecules/ng of DNA. In most DNA samples obtained from glioblastoma cells, the dosage of *IL-6* gene was found to be elevated, varying between 335 to 1135 gene molecules/ng of DNA (mean, 719 ± 295). The *IL-6* gene copy-number ratio of tumour DNA to normal DNA varied from 1.58 to 4.87 (Table 1).

As the increase in *IL-6* gene dosage was relatively low, we developed a sensitive real-time PCR strategy to confirm the findings of competitive PCR. The real-time PCR analysis demonstrated levels of *IL-6* gene over-representation that were similar to those obtained with competitive PCR (Table 1). A highly significant correlation was found between the results of two methods ($r = 0.94$, $P < 0.001$). Interestingly, according to the results of both methods, *IL-6* gene over-representation was higher in primary glioblastoma cells than in cell lines.

To investigate the possibility that the increased dosage of IL-6 gene on 7p was due to a gain of the whole chromosome 7, we determined the dosage of *CFTR* gene located on chromosome 7q. As shown in Figure 3, the dosage of *CFTR* gene in tumour DNA was elevated approximately 1.6-fold in GBM1 and GBM3

© 2001 Cancer Research Campaign 

**Table 1** *IL-6* gene dosage and mRNA level in glioblastoma cells. The data are presented as mean ± SD of experiments performed in triplicate

| Glioblastoma specimens | Fold-increase in IL-6 gene dosage | | *IL-6* mRNA level | |
|---|---|---|---|---|
| | Competitive PCR (tumour/normal DNA copy-number ratio) | Real-time PCR (*IL-6*/beta globin copy-number ratio) | Relative expression rate *IL-6*/β2 microglobulin | Real-time RT-PCR *IL-6*/18S (Δ Δ $C_T$) |
| Primary cells | | | | |
| GBM2 | 4.87 ± 0.13 | 4.29 ± 0.08 | 0.90 ± 0.07 | 14.95 ± 2.61 |
| GBM5 | 4.70 ± 0.17 | 4.89 ± 0.10 | 1.06 ± 0.10 | 60.50 ± 5.28 |
| GBM1 | 4.03 ± 0.27 | 3.99 ± 0.06 | 0.05* | 0.04 ± 0.03 |
| GBM4 | 3.06 ± 0.10 | 3.19 ± 0.15 | 0.26 ± 0.05 | 0.12 ± 0.03 |
| GBM3 | 2.89 ± 0.10 | 3.31 ± 0.08 | 0.23 ± 0.06 | 0.59 ± 0.01 |
| Cell lines | | | | |
| SF763 | 2.86 ± 0.11 | 2.90 ± 0.04 | 0.68 ± 0.09 | 8.14 ± 0.32 |
| U251 | 2.56 ± 0.04 | 3.14 ± 0.11 | 0.40 ± 0.004 | 1.00 ± 0.11** |
| U87 | 1.96 ± 0.02 | 2.28 ± 0.12 | 0.86 ± 0.09 | 20.01 ± 3.43 |
| G5 | 1.58 ± 0.07 | 1.53 ± 0.05 | 0.75 ± 0.01 | 7.48 ± 3.45 |
| SF767 | 1.17 ± 0.08 | 1.23 ± 0.03 | 0.00 | 0.00 |

*Weak *IL-6* mRNA expression at the limit of detection; **U251 cells were used as a calibrator for *IL-6* mRNA.

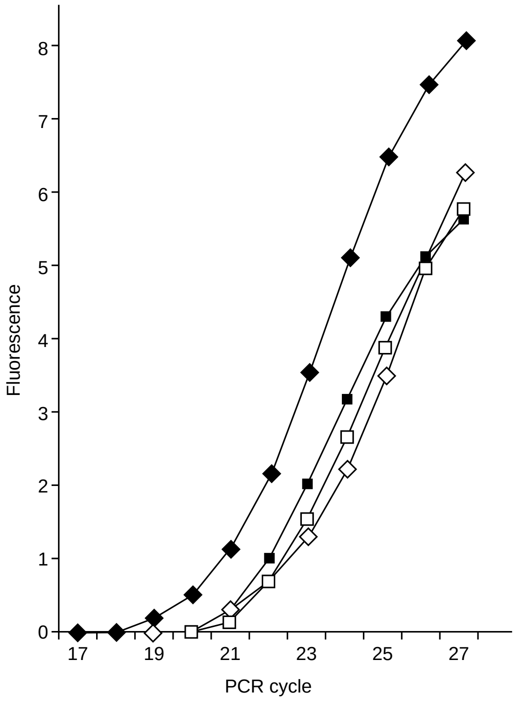

| Sample/gene | $C_T$ | Copy number |
|---|---|---|
| GBM5 DNA/IL-6 | 18.80 | 63,207 |
| Normal DNA/IL-6 | 20.30 | 12,184 |
| GBM5 DNA/beta-globin | 20.35 | 12,933 |
| Normal DNA/beta-globin | 20.43 | 12,367 |

**Figure 2** Amplification profiles for *IL-6* and beta-globin genes obtained on the LightCycler from equal amounts of glioblastoma tumour GBM5 and normal DNA. The fluorescence (SYBR Green I) is depicted on the Y axis; X-axis shows the number of PCR cycles. Quantitation of copy numbers was based on determination of the crossover point ($C_T$), marking the cycle when fluorescence of a given sample rose above the background level

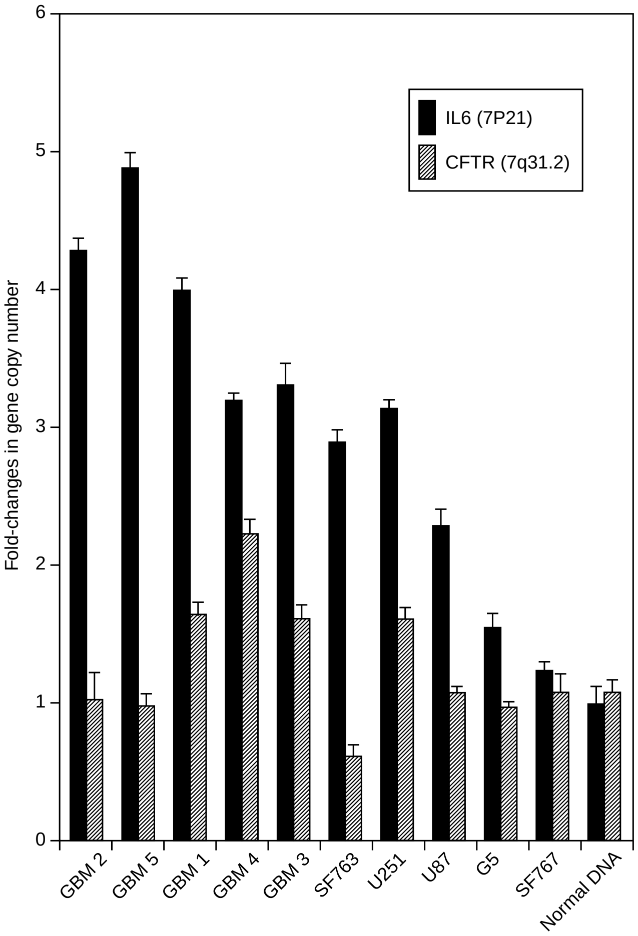

**Figure 3** Fold-changes in the dosage of *IL-6* gene (chromosome 7p) and *CFTR* gene (7q) estimated in glioblastoma and normal DNA samples relative to a reference gene, the beta-globin (chromosome 11p)

tumours, and in U251 cell line, suggesting a trisomy 7; and 2.2-fold in the GBM4 tumour, indicating either a tetrasomy 7 or a low-level amplification involving 7q31.2 chromosomal region. The SF763 cell line demonstrated an underrepresentation of *CFTR* gene of about 50%. *CFTR* gene dosage was found to be identical to that of normal DNA in the remaining primary tumours and cell lines. Overall, the dosage of *IL-6* gene was higher than that of *CFTR* gene in most glioblastoma samples.

© 2001 Cancer Research Campaign

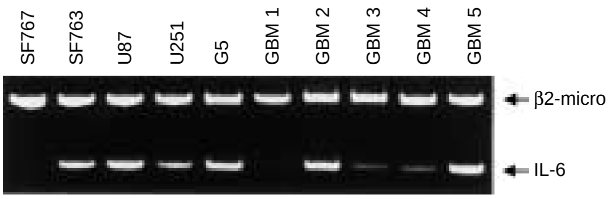

**Figure 4** RT-PCR results for *IL-6* and β2-microglobulin mRNA obtained in glioblastoma cell lines and primary cells

### IL-6 gene expression

*IL-6* expression at the RNA level was studied using two quantitative RT-PCR-based methods (Table 1). The results of two techniques, RER *IL-6*/β2-microglobulin and real-time PCR, correlated well ($r = 0.98$, $P < 0.001$, log values of real-time PCR data were used). Furthermore, we have found, for the primary glioblastoma tumours included in the present study, a good concordance between RNA expression (RER *IL-6*/β2-microglobulin) and protein detection using semiquantitative *IL-6* immunohistochemistry of neoplastic cells (Rolhion et al, 2001). Among primary tumour samples, the highest levels of *IL-6* expression were detected in two samples, GBM2 and GBM5, that harboured the highest *IL-6* gene copy number. However, GBM1 tumour with a 4-fold overrepresentation of the *IL-6* gene showed a weak expression of *IL-6* mRNA. All but one tumour cell line showed substantial levels of *IL-6* mRNA (Figure 4). No *IL-6* expression was detected in the SF767 cells that had no *IL-6* gene over-representation.

### DISCUSSION

The studies described in this paper were initiated in order to estimate the number of *IL-6* gene copies in primary cells and cell lines derived from glioblastoma tumours, which frequently show *IL-6* over-expression. Using competitive PCR, we found that the number of *IL-6* gene copies was increased 3- to 5-fold in 5 of 5 primary tumour samples and 1.5- to 3-fold in 4 of 5 glioblastoma cell lines. This finding was confirmed by means of real-time PCR which has been proven to be the most appropriate method for detecting low-level (≤ 5-fold) gene amplifications (Bièche et al, 1998). The number of *IL-6* gene copies as determined by both methods matched very well.

The overrepresentation of *IL-6* gene sequences detected in our series of glioblastomas most likely results from amplifications involving the *IL-6* gene locus on chromosome 7p21. This hypothesis is supported by the observation that large amplicons on chromosome 7p are present in approximately two-thirds of human glioblastomas (Harada et al, 1998). It seems unlikely that the 3- to 5-fold increase in *IL-6* gene dosage results from gains of the whole chromosome 7. When the copy number of *CFTR* gene from chromosome 7q was quantitated, a 1.5-fold increase as compared with normal DNA was found in 3 cases, suggestive of trisomy 7, and a 2.2-fold increase only in 1 case. In most individual cases of glioblastoma tumours and cell lines, the representation of *IL-6* gene DNA was obviously higher than that of *CFTR* gene. Although the number of tumours analysed in our study was small, a high frequency of *IL-6* amplification – 9 out of 10 cases – suggests that this genetic alteration may be a common feature in glioblastomas.

Amplification may be one of mechanisms mediating the upregulation of *IL-6* gene in glioblastomas. In our study, all glioblastomas with amplified *IL-6* gene expressed *IL-6* mRNA. Interestingly, a higher level of expression was found in tumours with the highest gene copy number, as measured using two different quantitative RT-PCR approaches. However, no clear correlation could be discerned between *IL-6* gene copy number and *IL-6* mRNA level in the whole series. Similar observation was reported in breast tumours where *MYC* gene over-expression did not correlate with DNA over-representation at the *MYC* locus (Bièche et al, 1999). In the same way, MDM2 amplification and overexpression did not correlate in adult soft tissue sarcoma (Cordon-Cardo et al, 1994). The amplified genes may be rearranged, like the *EGFR* gene in some glioblastoma tumours (Ekstrand et al, 1992), and therefore may not be over-expressed. These findings are consistent with the idea that not only the increase in gene copy number itself but also changes in the amplicon structure regulate the mRNA expression.

Amplifications of several genes that are implicated in tumour progression characterize glioblastomas (see for review Pathology and Genetics of Tumours of the Nervous System, 2000). In tumour cells, the amplicons containing *EGFR*, the gene the most frequently amplified in glioblastomas (Canute et al, 1996), *MET* (Fischer et al, 1995), *c-MYC* (Trent et al, 1986) and *GLI* (Xiao et al, 1994) are located on double minute chromosomes (DMs). These observations indicate that glioblastomas are susceptible to gene amplifications manifesting as DMs. It is reasonable to hypothesize that *IL-6* amplicons are present in glioblastomas on DMs. Indirect evidence for this localization may be provided by the observation that primary tumour cells revealed higher levels of *IL-6* amplification than glioblastoma cell lines. As DMs are randomly distributed to daughter cells during division, the change in selection pressure when glioblastoma cells are grown in vitro could reduce the frequency of DMs. We envisage studies using fluorescence in situ hybridization (FISH) with *IL-6*-gene specific molecular probes to confirm and examine *IL-6* gene amplification and its localization. The cell-by-cell analysis using FISH is the most suitable method for this purpose, as amplifications on chromosome 7p in glioblastomas are intratumorally heterogeneous and in some tumours only found in single areas (Jung et al, 1999).

*IL-6* expression appears to be a marker of glioma malignancy that is strongly correlated with tumour aggressiveness and could be considered as a potential therapeutic target. In this regard, further studies of *IL-6* gene amplification and amplicon localization on large series of primary tumours are essential to gain information that may be of value for new therapeutic investigations.

### ACKNOWLEDGEMENTS

This work was supported by the Ligue Nationale Contre le Cancer (Comité du Puy de Dôme). We are grateful to Anne Cayre and Annie Dosgilbert for their assistance in *IL-6* expression measurements.

### REFERENCES

Bièche I, Olivi M, Champeme MH, Vidaud D, Lidereau R and Vidaud M (1998) Novel approach to quantitative polymerase chain reaction using real-time detection: application to the detection of gene amplification in breast cancer. *Int J Cancer* **78**: 661–666

Bièche I, Laurendeau I, Tozlu S, Olivi M, Vidaud D, Lidereau R and Vidaud M (1999) Quantitation of MYC gene expression in sporadic breast tumors with a real-time reverse transcription-PCR assay. *Cancer Res* **59**: 2759–2765

Borsellino N, Belldegrun A and Bonavida B (1995) Endogenous interleukin 6 is a resistance factor for cis-diamminedichloroplatinum and etoposide-mediated cytotoxicity of human prostate carcinoma cell lines. *Cancer Res* **15**: 4633–4649

© 2001 Cancer Research Campaign 

Buronfosse A, Thomas CP, Ginestet C and Doré J-F (1994) Radiosensitivity in vitro of clonogenic and non-clonogenic glioblastoma cells obtained from a human brain tumor. *C R Acad Sci Paris, Sciences de la vie/Life sciences* **317**: 1031–1047

Canute GW, Longo SL, Longo JA, Winfield JA, Nevaldine BH and Hahn PJ (1996) Hydroxyurea accelerates the loss of epidermal growth factor receptor genes amplified as double-minute chromosomes in human glioblastoma multiforme. *Neurosurgery* **39**: 976–983

Celi F, Zenilman M and Shuldiner A (1993) A rapid and versatile method to synthesize internal standards for competitive PCR. *Nucleic Acid Res* **21**: 1047

Comparative $C_T$ method in ABI PRISM 7700 Sequence Detection System (1997) In: *PE Applied Biosystems User Bulletin #2* pp 11–24

Cordon-Cardo C, Latres E, Drobnjak M, Oliva MR, Pollack D, Woodruff JM, Marechal V, Chen J, Brennan MF and Levine AJ (1994) Molecular abnormalities of mdm2 and p53 genes in adult soft tissue sarcomas. *Cancer Res* **54**: 794–799

Dean M, White MB, Amos J, Gerrard B, Stewart C, Khaw KT and Leppert M (1990) Multiple mutations in highly conserved residues are found in mildly affected cystic fibrosis patients. *Cell* **61**: 863–870

Ekstrand AJ, Sugawa N, James CD and Collins VP (1992) Amplified and rearranged epidermal growth factor receptor genes in human glioblastomas reveal deletions of sequences encoding portions of the N-and/or C-terminal tails. *Proc Natl Acad Sci USA* **89**: 4309–4313

Fischer U, Muller HW, Sattler HP, Feiden K, Zang KD and Meese E (1995) Amplification of the MET gene in glioma. *Genes Chromosomes Cancer* **12**: 63–65

Goswami S, Gupta A and Sharma SK (1998) Interleukin-6-mediated autocrine growth promotion in human glioblastoma multiforme cell line U87MG. *J Neurochem* **71**: 1837–1845

Harada K, Nishizaki T, Ozaki S, Kubota H, Ito H and Sasaki K (1998) Intratumoural cytogenetic heterogeneity detected by comparative genomic hybridization and laser scanning cytometry in human gliomas. *Cancer Res* **58**: 4694–4700

Joy A, Moffett J, Neary K, Mordechai E, Stachowiak E, Coons S, Rankin-Shapiro J, Florkiewicz RZ and Stachowiak MK (1997) Nuclear accumulation of FGF-2 is associated with proliferation of human astrocytes and glioma cells *Oncogene* **14**: 171–183

Jung V, Romeike BF, Henn W, Feiden W, Moringlane JR, Zang KD and Urbschat S (1999) Evidence of focal genetic microheterogeneity in glioblastoma multiforme by area-specific CGH on microdissected tumor cells. *J Neuropathol Exp Neurol* **58**: 993–999

Miwa H, Kanno H, Munakata S, Akano Y, Taniwaki M and Aozasa K (2000) Induction of chromosomal aberrations and growth-transformation of lymphoblastoid cell lines by inhibition of reactive oxygen species induced apoptosis with interleukin 6. *Lab Invest* **80**: 725–734

Nishizaki T, Ozaki S, Harada K, Ito H, Arai H, Beppu T and Sasaki K (1998) Investigation of genetic alterations associated with the grade of astrocytic tumor by comparative genomic hybridization. *Genes Chromosomes Cancer* **21**: 340–346

*Pathology and Genetics of Tumours of the Nervous System* (2000) Kleihues P, Cavenee WK (eds) pp. 9–52. IARC *Press*: Lyon

Rolhion C, Penault-Llorca F, Chevillard S, Verrelle P and Finat-Duclos F (1999) Quantification of RT-PCR products: assessment of ethidium bromide stained gel analysis in comparison with fluorescent detection using an automated sequencer. *Lab Med* **30**: 419–422

Rolhion C, Penault-Llorca F, Kemeny J-L, Lemaire J-J, Jullien C, Labit-Bouvier C, Finat-Duclos F and Verrelle P (2001) Interleukin-6 overexpression as a marker of malignancy in human glioma. *J Neurosurg* **94**: 97–101

Schlegel J, Scherthan H, Arens N, Stumm G and Kiessling M (1996) Detection of complex genetic alterations in human glioblastoma multiforme using comparative genomic hybridization. *J Neuropathol Exp Neurol* **55**: 81–87

Trent J, Meltzer P, Rosenblum M, Harsh G, Kinzler K, Mashal R, Feinberg A and Vogelstein B (1986) Evidence for rearrangement, amplification, and expression of c-myc in a human glioblastoma. *Proc Natl Acad Sci USA* **83**: 470–473

Van Meir E, Sawamura Y, Diserens A-C, Hamou M-F and de Tribolet N (1990) Human glioblastoma cells release interleukin 6 in vivo and in vitro. *Cancer Res* **54**: 649–652

Van Meir EG, Kikuchi T, Tada MLiH, Diserens AC, Wojcik BE, Huang H-JS, Friedmann T, de Tribolet N and Cavenee WK (1994) Analysis of the p53 gene and its expression in human glioblastoma cells. *Cancer Res* **54**: 649–652

Weber RG, Sommer C, Albert FK, Kiessling M and Cremer T (1996) Clinically distinct subgroups of glioblastoma multiforme studied by comparative genomic hybridization. *Lab Invest* **74**: 108–119

Xiao H, Goldthwait DA and Mapstone T (1994) A search for gli expression in tumours of the central nervous system. *Pediatr Neurosurg* **20**: 178–182

Yasukawa K, Hirano T, Watanabe Y, Moratanik, Matsuda T, Nakai S, Kishimoto T (1987) Structure and expression of human B cell stimulatory factor-2 (BSF-2/IL-6) gene. *EMBOJ* **6**: 2939–2945

 *© 2001 Cancer Research Campaign*
